# Comparison of virtual reality to physical box and blocks on cortical an neuromuscualar activations in young adults

**DOI:** 10.1038/s41598-023-43073-2

**Published:** 2023-10-02

**Authors:** Sheridan M. Parker, Brian Ricks, Jorge Zuniga, Brian A. Knarr

**Affiliations:** 1 Department of Biomechanics, University of Nebraska at Omaha, 6160 University Dr S., Omaha, NE 68182 USA; 2 Department of Computer Science, University of Nebraska at Omaha, 1110 South 67th Street, Omaha, NE 68182 USA

**Keywords:** Somatosensory system, Control theory, Electromyography - EMG, Near-infrared spectroscopy

## Abstract

The purpose of this study was to assess the changes in neural activations when performing the box and block test (BBT) in virtual reality (VR) compared to the physical BBT. Young healthy participants performed three trials of the BBT with their left and right hands in both the VR BBT, using VR hand controllers, and physical BBT conditions. Electromyography sensors were placed on the upper extremity of both arms and functional near-infrared spectroscopy was used to measure motor cortex activations throughout each condition. While a reduction in BBT score and increased wrist extensor neuromuscular activity is exhibited during the VR condition, there is no statistical difference in motor cortex activation between the two BBT conditions. This work provides a basis for exploring cortical and neuromuscular responses to VR in patient populations.

## Introduction

### Neurorehabilitation

Clinical neurorehabilitation is focused on intensive repetitive motions that target simple actions to improve motor functions for individuals that experience nervous system disorders. Specifically, neurorehabilitation combines neuroscience and biomechanics research to understand recovery and compensatory mechanisms related to patients’ specific impairments. The goal of neurorehabilitation is to maximize functional movements, community participation, and quality of life^1^. Rehabilitation often includes interdisciplinary programs that establish a set of goals and specific needs of the patient to develop measures that assist the patient to reach their optimal functional ability, maximize independence and regain community engagement^2^. The interdisciplinary aspect of these programs often allows them to be tailored to the patients’ specific needs, whether that is a focus on the upper extremity, lower extremity, balance, or any other multitude of functional domains.

Neurorehabilitation often covers three domains: basic conventional therapy approaches, adjunct therapies, and complications management^3^. Conventional therapy is defined as behavioral therapy directed by a clinician and follows standardized guidelines and practices^3^. For the purpose of this study, we will focus on one common rehabilitation tool from conventional therapy, the box and block test (BBT). The goal of the BBT is to move as many 1-in. blocks from one box over a partition to the other box as fast as possible within a 1-min time limit. This is a common measure of gross hand dexterity that can provide information about upper extremity functional ability in a quick and engaging manner. Even though conventional rehabilitation covers multiple domains of function and is the most widely utilized form of neurorehabilitation, there are still major limitations. With 3.8 millions of Americans with chronic health problems experiencing limited access to transportation, regular clinical rehabilitation visits can be inconvenient and expense^4–8^. Conventional therapy is also associated with self-directed home exercises that lack feedback and can be monotonous, leading to a decrease in motivation and natively impact high dose intensity therapy need^4^. Therefore, there is a need for more engaging, home-based therapy that provides valuable feedback.

Adjunct therapies are meant to assist and enhance conventional therapy approaches and can include a variety of methods including mental practice, non-invasive brain stimulation, and virtual reality (VR)^3^. These adjunct therapies often are not as well standardized as conventional therapy but still demonstrate promising results for improving functional outcomes for patients^3^. Previous literature has shown that by increasing motivation and engagement, with encouraging exercises, rehabilitation outcomes can improve for clinical populations^4^. These adjunct therapies can be developed in a variety of ways that improve patient engagement, motivation, and adherence to rehabilitation both within the clinic and in home-based settings.

### Virtual reality for neurorehabilitation

A way to address the limitations of conventional therapy is through the use of VR technology. VR allows for the development of custom games that can direct specific movements as a basis for neurorehabilitation and as an adjunct therapy. Previous literature have presented VR in a multitude of ways from being screen based to immersive with different ways of interacting with the virtual environment from camera-based motion sensing to hand held controllers^9–13^. The flexibility of VR systems allows for these systems to be implemented in various clinical and home-based settings while using less physical space than traditional rehabilitation. This allows for VR to assist in rehabilitation programs by providing directed highly intensive repetitive motions in a variety of settings.

Pervious literature has investigated the use of VR systems on functional outcomes for clinical populations. A meta-analysis found that there is no statistical difference in functional outcome measures, Mini Mental Scale Examination (MMSE) and Fugl–Meyer score between patients that received VR-based or conventional therapy and concluded that that rehabilitation programs that incorporate VR are associated with improved outcomes but not statistically difference compared to conventional rehabilitation^14,15^. Previous literature has also found that VR-based rehabilitation can increase upper-extremity measures of functionality, specifically through the Action Research Arm test, Wolf-Motor Function Test, grip strength, and BBT scores^16,17^. A study by Stockley et al.^18^, found that clinicians initially had concerns about using VR-based rehabilitation but the concerns decreased with training, reference materials and technical support. The authors also found that the clinicians indicted a higher functional task carry-over ability and higher patient engagement levels when using the VR-based rehabilitation^18^. This indicates that VR can provide similar functional outcomes while also addressing some limitations of conventional rehabilitation.

However, many of these studies have used basic reaching and grasping tasks on a 2D computer screen to emulate the virtual environment. An advantage of VR is the ability to design and develop custom games that target patient specific needs. There are two main types of VR games available exergames, which are commercially available games that can be adapted for rehabilitation purposes, and serious games, which are designed specifically for rehabilitation needs^4^. A study by Gustavsson et al.^19^ investigated participant’s perceptions on using exergames during a 10-week VR intervention and found that participants had positive perceptions about the games being motivating, the importance of getting feedback, impact on daily life, and changes in difficulty during the intervention. The BBT is a common rehabilitation tool that measures gross hand dexterity and has recently been developed as a VR serious game^20–24^. A study by Everard et al. concluded that the virtual BBT serious game is usable, valid, and reliable to use with both healthy and individuals with stroke^23^. Another study found that home-based therapy with games, regardless if they were serious or exergames, increased therapy participation, increased activities of daily living independence, and gave the participants a sense of ownership over their rehabilitation^4^. The application of serious and exergames in VR has been well received by clinical populations and practitioners and has been shown to improve functional outcomes. While functional outcomes have been widely reported when using VR for neurorehabilitation, the “neuro” aspect of neurorehabilitation during VR use is not well understood with limited reporting.

### Neuroimaging to assess rehabilitation improvements

By understanding the “neuro” aspect of neurorehabilitation, rehabilitation tools can be better defined for patient-specific needs. A study by Thirumala et al.^25^ concludes that by examining specific rehabilitation protocols, like VR, the cortical and sub-cortical mechanisms that drive these outcomes can be explained. Functional neuroimaging of the brain has been proven as a useful tool for understanding the cortical and sub-cortical mechanisms and pathways that mediate motor recovery and neural plasticity associated with rehabilitation^25–27^. Włodarczyk et al.^27^ argues that neuroimaging techniques may provide descriptions of the mechanisms that could be used as quantitative indicators for the mechanistic response to neurorehabilitation. Although to fully understand the neural mechanistic response in clinical populations for VR, understanding normal patterns of activations within neurologically intact control populations is necessary for interpreting the impact of VR on neurorehabilitation outcomes^27^. There are several neuroimaging techniques available that can be used to accomplish this goal from the gold-standard functional magnetic resonance imaging, to electroencephalography, to functional near-infrared spectroscopy (fNIRS).

fNIRS measures the changes in cortical blood oxygenation by detecting differences in the absorption of near-infrared light between oxygenated hemoglobin (HbO) and deoxygenated hemoglobin (HbR) concentrations based on the principle of neurovascular coupling^28,29^. This principle states that as neural activations occur there is an increase in cerebral blood flow (CBF) which leads to an increase in HbO and a washout of HbR due to a lack of nutrient reserves in the brain^30–34^. fNIRS uses this principle of neurovascular coupling by measuring the local changes in hemodynamic response to infer neuronal activity. To detect hemodynamic response changes, fNIRS systems use light wavelengths between 600 and 2500 nm, the NIRS spectral window, to probe human tissue which is transparent within this wavelength window^32,33^. The optimal range to investigate in-vivo optical properties is 650–950 mm due to the scattering properties of human tissue^33^. When NIRS light penetrates these tissues, light photons are either absorbed by hemoglobin chromophores or scattered. This attenuation of light intensity is recorded by detector sensors and used to calculate HbO, HbR, and total hemoglobin (HbT) concentrations^30–34^. Much like functional magnetic resonance imaging that relies on the blood oxygen level dependent (BOLD) signal, a similar neurovascular coupling signal, fNIRS has emerged as a practical neuroimaging technique to assess hemodynamic cortical responses and cortical reorganization.

There are several advantages and disadvantages to using fNIRS systems. fNIRS systems have limited clinical applicability due to lower spatial specificity, and lower temporal resolution^30,32,34^. The spatial specificity is limited by the number of sensor-detector optodes but can be improved by increasing the optode density^32,34^. Even with increased optode density, NIR light probing is limited to the cortical layer of the brain therefore deeper brain structure activations are unreachable^30^. fNIRS systems are also limited by lower inter-subject reproducibility due to skull thickness, dura mater thickness, hair density, hair color, ambient light, and optical heterogeneity within the head^34^. However, by delivering near-infrared light via fiber optic cables, fNIRS is less sensitive to noise and movement artifacts compared to functional magnetic resonance imaging, the gold standard for brain imaging, which makes fNIRS easier for individuals to tolerate during movement tasks^28,29^. fNIRS systems are non-invasive and more portable compared to other neuroimaging modalities allowing for a wider range of static and dynamic tasks to be performed with multiple participant populations from infants to clinical populations. Although how the brain reacts to immersive virtual reality and clinically based games remains not well known.

### Study purpose and hypothesis

The purpose of this study is to assess the changes in neural activations when performing the BBT in VR compared to the physical BBT. We hypothesize that there will be no difference in whole motor cortex activation between the physical and VR BBT conditions for both the left and right hands. The BBT performed in VR is clinically driven meaning that the task is similar to the physical BBT commonly used in rehabilitation. Therefore, the functional task will be the same between the two conditions and the difference in mode will have no effect on motor cortex activations. We further hypothesize that there will be an increase in peak EMG activation exhibited during the VR BBT condition compared to the physical BBT condition for both the left and right arms. The BBT in VR is assumed to be a novel task which inherently has a reduction in somatosensory input from grasping and object manipulation with the VR hand controllers. This will cause an increase in peak EMG activation of the upper extremities.

## Results

### Box and block test scores

**Figure 1:**
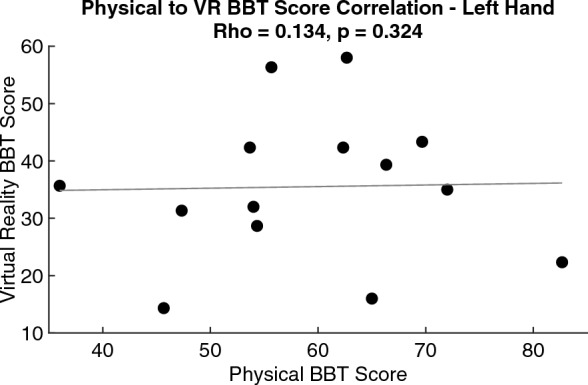
Correlation of physical BBT score to VR BBT score for the left hand. There is no significant relationship between the two environments on BBT score.

**Figure 2:**
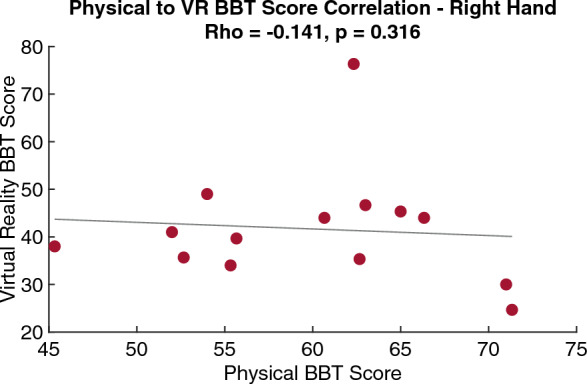
Correlation of physical BBT score to VR BBT score for the right hand. There is no significant relationship between the two environments on BBT score.

An average of 24 and 18 more blocks were moved during physical condition compared to the VR BBT condition for the left and right hands, respectfully. Participants scored higher during the physical BBT condition compared to the VR BBT condition for both the left (p = 0.002) and right (p = 0.002) hands (Table 1). There was no significant relationship between the VR and physical BBT scores for both the left (rho = 0.134, p = 0.324, Fig. 1) and right hands (rho = − 0.141, p = 0.316, Fig. 2).

**Table 1:** Comparison of BBT scores (mean ± standard deviation).

Hand	Physical BBT score	VR BBT score	Statistical significance
Left hand (n = 14)	59 ± 12	36 ± 13	p = 0.002
Right hand (n = 14)	60 ± 8	42 ± 12	p = 0.002

### Motor cortex activation

**Figure 3:**
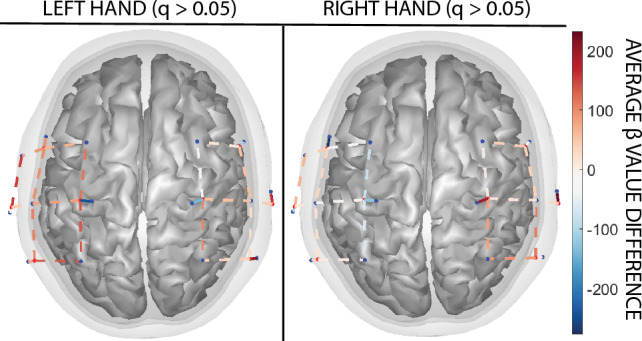
Comparison of motor cortex activations between the VR and physical BBT conditions for the left (left image) and right (right image) hands. There is no statistical difference between the two conditions on motor cortex activations for either hand.

There was no statistical difference in motor cortex activation between the VR and physical conditions for both the left (all channels: q > 0.05) and right (all channels: q > 0.05) hands (Fig. 3, Table 2).

**Table 2:** Average beta (β) values and t-statistics for each sensor-detector pair for each hand between the physical and VR BBT conditions (n = 14).

Source	Detector	Right hand				Left hand			
		Beta value	Tstat	q	Power	Beta value	Tstat	q	Power
1	1	− 79.87	− 1.86	0.24	0.31	116.49	2.59	0.11	0.53
1	2	− 66.43	− 1.34	0.44	0.34	136.26	2.68	0.10	0.56
1	3	− 4.91	− 0.10	0.94	0.75	85.09	1.72	0.26	0.32
2	1	− 22.88	− 0.44	0.81	0.50	69.14	1.35	0.44	0.34
2	3	31.46	0.58	0.73	0.46	101.20	1.81	0.25	0.31
2	4	42.26	0.80	0.65	0.41	91.05	1.72	0.26	0.32
3	2	6.23	0.14	0.93	0.70	135.99	3.03	0.08	0.69
3	3	20.38	0.38	0.85	0.53	107.74	2.00	0.22	0.30
4	3	10.36	0.23	0.89	0.62	44.74	0.99	0.58	0.38
4	4	66.21	1.16	0.47	0.36	152.80	2.76	0.10	0.59
5	5	4.85	0.11	0.94	0.74	− 11.14	− 0.25	0.89	0.60
5	6	83.13	1.60	0.31	0.32	102.88	2.01	0.22	0.31
5	7	14.43	0.28	0.89	0.59	55.02	1.06	0.55	0.37
6	5	43.22	0.85	0.62	0.40	11.51	0.23	0.89	0.62
6	7	40.72	0.74	0.67	0.42	55.87	1.01	0.58	0.38
7	6	89.97	2.09	0.19	0.33	37.87	0.89	0.61	0.39
7	7	112.21	2.23	0.17	0.38	29.26	0.58	0.73	0.46
8	7	31.70	0.65	0.70	0.44	46.79	0.97	0.58	0.38

### Neuromuscular activations

**Figure 4:**
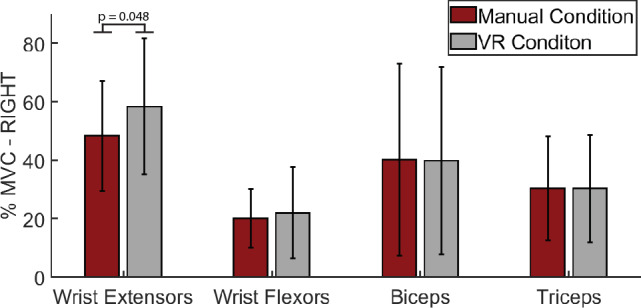
Comparison of peak neuromuscular activations between the physical BBT and VR BBT conditions for the right hand. The wrist extensors exhibited statistically increased activation during the VR condition. There was no statistical difference between environments on the wrist flexors, biceps, and triceps muscles.

Participants (n = 14) exhibited significantly higher activations during the VR BBT condition for the wrist extensors (VR: 58.37 ± 23.31%MVC, physical: 48.37 ± 18.81%MVC, p = 0.048) compared to the physical BBT condition for the right hand. There was no statistical difference between the VR BBT and physical BBT conditions in the wrist flexors (VR: 22.09 ± 15.51%MVC, physical: 20.13 ± 9.95%MVC, p = 0.925), biceps brachii (VR: 39.92 ± 32.01%MVC, physical: 40.20 ± 32.71%MVC, p = 0.331) and lateral triceps (VR: 30.32 ± 18.3%MVC, physical: 30.4 ± 17.80%MVC, p = 0.975) for the right hand (Fig. 4).

**Figure 5:**
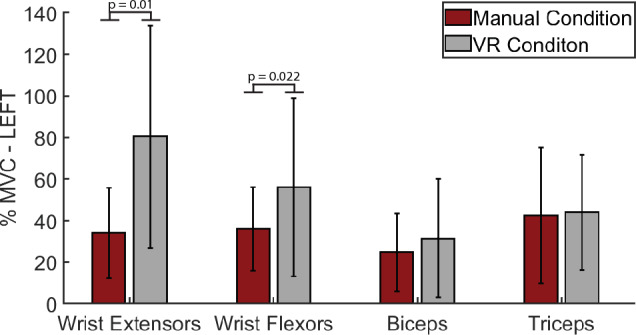
Comparison of peak neuromuscular activations between the physical BBT and VR BBT conditions for the left hand. The wrist extensors and flexors exhibited statistically increased activation during the VR condition. There was no statistical difference between environments for the biceps or triceps muscles.

Participants exhibited significantly higher activations during the VR BBT conditions for the wrist extensors (VR: 80.42 ± 53.59%MVC, physical: 34.04 ± 21.78%MVC, p = 0.01) and wrist flexors (VR: 56.09 ± 43.04%MVC, physical: 36.06 ± 19.99%MVC, p = 0.022) when performing the BBT with the left hand. There was no statistical difference between the physical and VR BBT conditions in the Biceps Brachii (VR: 31.51 ± 28.61%MVC, physical: 24.78 ± 18.78%MVC, p = 0.14) or Lateral Triceps (VR: 43.93 ± 27.67%MVC, physical: 42.49 ± 32.78%MVC, p = 0.683) for the left hand (Fig. 5).

## Discussion

The purpose of this study was to assess the changes in neural activations when performing the BBT in VR compared to the physical BBT. While we found a reduction in gross dexterity and an increase in neuromuscular activations during the VR BBT condition there was no statistical difference in motor cortex activations between the VR BBT and Physical BBT condition. Therefore, employing VR in neurorehabilitation may be beneficial for clinical populations, like stroke, which benefit from increased neuromotor training.

Participants moved more blocks during the physical condition compared to the VR BBT condition for both the left and right hands. Specifically, participants moved 24 blocks more for the left hand 18 blocks more for the right hand during the physical condition. Overall these values are lower than the established BBT norms for individuals in the same age-range^35^. While the results indicate that the participants are moving more during within the physical environment compared to the VR environment, there could be an effect of sensory input. The tactile and proprioceptive input from the blocks is lost during the VR BBT simulation, in which participants used VR hand controllers, so individuals must rely more on their visual inputs to understand where the blocks are and how to move them within the VR environment. This loss in sensory input could affect the motor output leading to a reduction in BBT score for the VR condition. This reduction could also be explained by a constraint of the VR BBT simulation. For the simulation to count a block as a point, the VR hand controller must pass completely over the box partition. The increased reliance on visual input and a decrease in tactile input could affect individual’s proprioception and their understanding of where the hand controllers are in the VR environment and thus a reduction in VR BBT score. This difference could be supported by the difference in neuromuscular activations.

Participants exhibited significantly increased wrist extensor activation for both hands while increased wrist flexor activations for the left hand when performing the BBT in VR compared to the physical condition. The increased activation during the VR condition could be a potential compensatory mechanism where individuals increase the wrist activations to compensate for the lack of tactile and proprioceptive input within the VR condition. The general strategy for the BBT, grabbing a block moving across the partition and release, is similar between the VR and physical environment. The dimensions of both BBT boxes are the same and centered in front of the participants which leads the task to be more forearm and shoulder dominant. Therefore, there would be less elbow flexion/extension which could elucidate the similarity in biceps and triceps activation between the VR and physical environmental conditions. Much like exercising, the increased neuromuscular activation during the VR condition could lead to increased upper extremity strength and functionality. Interestingly even though we saw differences in both BBT scores and neuromuscular activations, there was no difference in motor cortex activation between environments. This indicates that the motor cortex is activating the same regardless of motor output.

There was no statistical difference in motor cortex activation between the VR and physical condition for both the left and right hands. In neurorehabilitation, one would assume that the “neuro” aspect would dictate the motor output; however, the motor cortex activations of this study refute this assumption. An explanation could be the importance of sensorimotor input for motor output. Applying the motor programming (schema) theory which states that hard wired neural connections, called motor programs, control task actions, like reaching for a block^36^. Due to the constraints of the head mounted display, we were only able to collect motor cortex activations regarding the activations of movement only. The difference in sensory between the VR and physical environments could affect the participant’s recalled motor programs without affecting the level of activation of the motor cortex leading. This would lead to the difference in BBT scores and neuromuscular activations between the VR and physical environments while not changing the motor cortex activations as the differences in brain activations could be located within the sensorimotor areas instead. These results have important clinical implications in that they suggest that VR neurorehabilitation tasks could be beneficial for individuals that require increased repetitive tasks.

While bare-hand tracking has become popular in VR applications, a lot of the studies use the LeapMotion system or Xbox Kinect to track bare hands. While these systems are able to track fine motion better than VR hand controllers, these systems must include additional integration equipment to the VR setup which could be cumbersome or confusing for clinicians and participants. A major goal in the development of the VR BBT simulation was that the set up would be as commercially available as possible therefore a potential clinical or user would just have to download the BBT simulation similar to downloading a video game without the additional tracking equipment.

The BBT is focused on assessing gross motor dexterity, meaning larger hand and arm movements. In terms of our setup, we are more interested in the gross motor dexterity that the BBT assess than the fine motor movements of finger grasping. Therefore, using the VR hand controllers is better for this task than using a bare-hand tracking system. We believe that the implicit tactile feedback of the VR controllers is more beneficial as the participants are able to press the controller trigger to pick up and have that tactile input that an object is grasped. Grasping with bare hands may not provide enough information about object manipulation in the context of this task.

There are several limitations to this study. The first limitation is the limited sample size. With a larger sample size some of the outcome measures might have a main effect that was not present in the current participant group. Another limitation of this study is that only the motor cortex activations could be collected due to the constraints of the VR head mounted display. While there were no changes that we detected within the motor cortices there could be differences in the occipital or sensorimotor areas of the brain that we were not able to detect. These potential changes in the occipital or sensorimotor areas could potentially account for the downstream effects in the BBT scores or neuromuscular activations that we can only allude to. A final limitation to this study is that we did not control VR familiarity. A participant that has more familiarity playing VR could perform better than a participant that is less familiar which could influence the results.

Future studies are needed to further understand the effect of immersive VR on brain and neuromuscular activations in clinical populations. Clinical populations, specifically neurologically impaired populations will respond to the immersive VR and clinically based VR simulations differently than neurologically intact populations will. As the goal of this simulation is to be used as an adjunct therapy, it will be beneficial to understand how the clinical population responds. Another future direction is the needed to understand sensory input effects. The results indicate that there may be differences in sensory input due to differences between the blocks and VR controllers. Therefore, understanding how sensory input, either tactile or visual, can be modulated would be beneficial. This study provides a basis for understanding the neural activations associated with VR serious games that focus on neurorehabilitation and provides a comparison for future studies to understand the effect of VR-based neurorehabilitation on clinical populations.

## Materials and methods

### Participant demographics

Twenty-four young healthy individuals participated in the study. Due to poor EMG recordings 7 participants were excluded from data analysis and a further 3 participants were excluded due to poor fNIRS calibrations. Data analysis was performed on 14 participants (7 male/7 female, age: 25 ± 3.82 year’s old, handedness: 2 left-/12 right-handed). All participants signed an approved informed consent form, and the study was approved by the University of Nebraska Medical Center Institutional Review Board. All study procedures were performed in accordance with the University of Nebraska Medical Center Institutional Review Board guidelines and regulations. The reference number for this study is IRB0904-20-EP.

The inclusion criteria included between the ages of 19–80 years old. The exclusion criteria included prior diagnosis of upper limb dysfunction or neurological disorders, musculoskeletal injuries in the upper limbs that resulted in surgery, medical conditions that would not allow for the use of the virtual reality headset or controllers, and/or a self-reported history of vertigo or inner ear disorders.

### Data collection

#### Physical box and block test

The physical BBT was placed at the midline of the participant on top of a table (height 0.76 m). The BBT consists of 150 wooden cubes (2.54 cm^3^). A 10 cm high partition placed at the center separates the wood box into two 25.4 cm square boxes. The participants were provided with the following instructions:

1. Move as many of the 1-in. blocks over the partition to the other side as fast as possible within the 1-min time limit.
2. Your hand must pass all the way over the partition for the block to count towards the score.
3. Only one block can be grabbed each time. If multiple blocks are grabbed, only one will be counted towards the score.
4. The blocks must land in the opposite box. If the block lands outside the box, it will not be counted towards the score.

#### Virtual reality system

The VR system (Oculus Rift, Oculus, Irvine, CA USA) is calibrated to the physical space with tracking sensors placed on either side of the computer monitor. A calibration height of 5′5″ within the Oculus device setup was used. This calibration height was chosen to ensure that the table height was standardized in the virtual space and physical environment at 0.76 m (standard table height) from the floor. The VR BBT simulation is centered on the mid-point of the VR headset and VR hand controllers were used to interact with the simulation. The participant remained sitting throughout the entire collection.

#### Virtual reality BBT simulation

**Figure 6:**
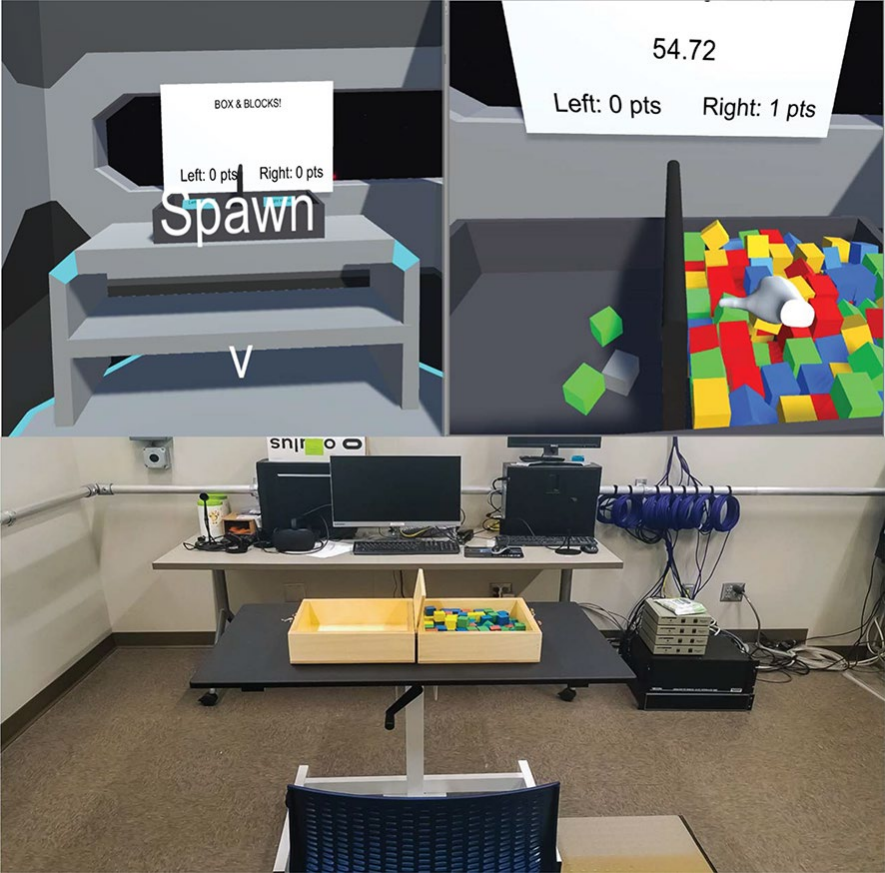
Comparison of the VR BBT simulation (top) setup to the physical BBT (bottom setup).

The VR BBT simulation was developed using the Unity platform (Unity Technologies, Austin TX, USA). Using Unity as the real-time simulation engine, the VR BBT simulation is completed using the Oculus Rift VR system on a 16 GB RAM Windows PC with an Intel Core i7-8700 processor. The BBT simulation features a set of logical constraints that align with the rules of the physical BBT. The virtual BBT is developed to have the same dimensions as the physical BBT within the virtual environment [53.7 cm (W) × 25.4 cm (H) × 8.5 cm (D)]. The virtual environment includes an enclosed room with 3 windows, a background made to look like a starry night, and a white scoreboard that displays a 1-min timer and BBT score directly in front of the user. The VR BBT simulation was developed in first-person perspective and the virtual avatar was represented as floating hands within the simulation space that the participants controlled (Fig. 6).

#### Functional near infrared spectroscopy (fNIRS)

**Figure 7:**
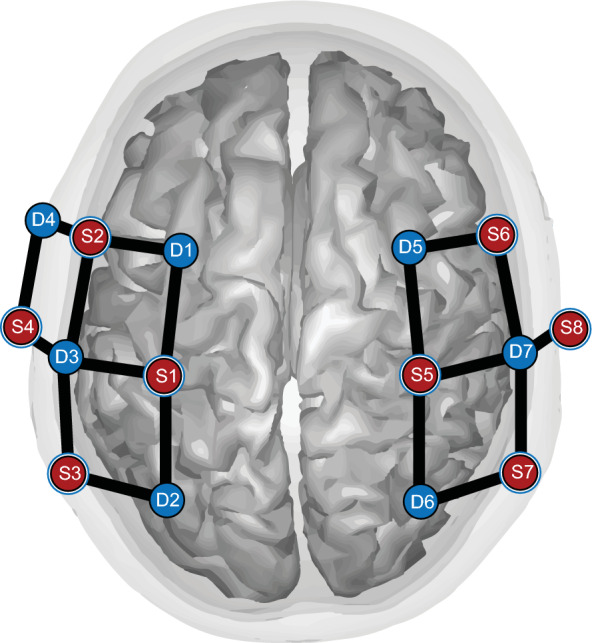
8 × 7 fNIRS sensor (red) and detector (blue) probe montage set up. The montage is centered over the Cz vertex with 10 measurement channels (black bars) over the left motor cortex and 9 measurement channels over the right motor cortex. Short-separation channels (blue rings) are attached to the sensor probes.

The Nirsport2 system (Nirx, Berlin, Germany) was used to measure brain activation of the left and right motor cortices. An 8 × 7 sensor-detector probe montage was centered around the CZ vertex and focused on the left and right motor cortices following the 10–20 international probe placement standard with a 30 mm inter-optode distance (Fig. 7). Eight (8) short separation channel probes were attached to the sensor probes with an inter-optode distance of 10 mm. Head circumference, Nasion (Nz) to Inion (IZ), and left pre-auricular (LPA) to right pre-auricular (RPA) distances were recorded to center the fNIRS cap and montage to the CZ. The fNIRS system recorded at a sampling frequency of 8.37 Hz.

#### Electromyography (EMG)

EMG sensors (Trigno Avanti, Delsys, Natick MA, USA) were placed bilaterally on the wrist flexors, wrist extensors, biceps brachii and lateral triceps. The sensor locations were determined by palpation and placed on the muscle bellies. Recording areas were cleaned and abrasion applied using alcohol prep wipes. EMG sensors were synched with the fNIRS system to produce a marker on the fNIRS signals at the start of EMG recordings. Sensor data was recorded at a sampling rate of 1200 HZ.

#### Collection procedures

**Figure 8:**
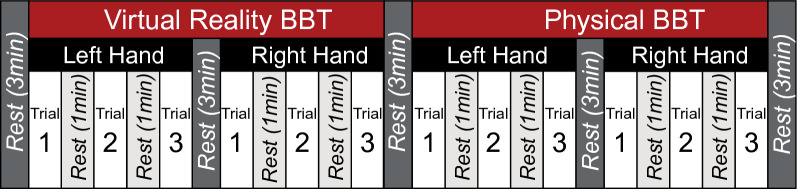
Schematic of the collection procedures. The VR BBT and Physical BBT conditions were randomized with the order of left and right hands further randomized within. Each trial lasted 1-min.

Participants first performed maximum voluntary contractions (MVCs) for each EMG sensor with a standard hand dynamometer (MicroFET2, Hoggan Scientific, Salt Lake City, UT USA). MVC collection consisted of 6 s maximum contractions with a 10 s rest for 3 trials. The participants were instructed to sit in a standardized position with their back against the seat, elbows resting on arm rests and elbows at 90° flexion. A crossover block design was used for this study where the participants were asked to perform the BBT for three (3) trials in VR and physically with their left and right hands (Fig. 8). The order of BBT modality (VR or physically) was randomized and the order of hands performed was further randomized. A total of twelve (12) trials were collected. Rests between trials were 1 min in duration and rests between conditions were 3 min in duration. fNIRS was used to measure levels of brain activation throughout each condition while EMG was used to measure neuromuscular activation during each trial.

### Data analysis

#### Box and block test scores

Means and standard deviation were calculated using BBT trial scores for physical BBT with the left hand, physical BBT with the right hand, VR BBT with the left hand and, VR BBT with the right-hand conditions.

#### Motor cortex activation

The motor cortex activations were calculated using a general linear model (GLM) analysis using the fNIRS sensor-detector data. The raw fNIRS signal were truncated with 30 s before the first trial and 30 after the last trial removed. The truncated raw signals were then resampled to 4 Hz to account for physiological noise. The resampled signals were then converted to optical density with a partial path length factor of 0.1. The optical density was then converted to oxygenated (HbO) and deoxygenated (HbR) hemoglobin concentrations using the modified Beer–Lambert Law. Short separation channel regression was applied to remove physiological noise. A GLM analysis was then applied with an auto-regressive iterative least squares (AR-ILS) processing module. The AR-ILS is a noise reduction filter method used for motion correction^37^.

The output of a GLM approach are beta (β) coefficients which are weighted values that transform the canonical hemodynamic response function (HRF) regressors into the measured signal. In context, these β coefficients are a measure of the amount of activation that is occurring in each measurement channel in reference to the expected canonical HRF. An increase in β coefficient would indicate an increase in motor cortex activation and thus an increase in upper extremity movement. Data analyses were performed in MATLAB 2018a (MathWorks, Natick MA, USA) using the NIRS Brain AnalyzIR toolbox^37^.

#### Neuromuscular activation

MVC signals were DC detrended to remove signal drift and center the signal around 0 v. The detrended signal was then filtered using 6th order Butterworth band-pass filter with cutoff frequency between 8 Hz and 95% power. The filtered signal was then full wave rectified and an 8 Hz low-pass linear envelope applied. The maximum value of the three MVC contracts were averaged and used as the MVC value for normalization for each sensor. The raw EMG task signals were DC detrended to remove signal drift and center the signal around 0v. The detrended signal was filtered using 6th order Butterworth Band-pass filter cutoff frequency between 8 Hz and 95% power. The filtered signal was then full wave rectified and a linear envelope applied. The linear envelope consisted of an 8 Hz low-pass 6th order Butterworth filter. The signal was then normalized to each sensor MVCs^38–42^. Signal peaks were then identified with a threshold of more than two standard deviations from the signal mean.

### Statistical analysis

#### Box and block test scores

The Shapiro–Wilk test was used to determine the normality of BBT scores. The right-hand VR BBT scores failed normality (p = 0.018) therefore two Wilcoxon signed rank tests were performed comparing the VR to physical BBT conditions for both the left and right hands. BBT scores was used as the variable of interest. To determine the relationship between VR to physical BBT scores, two two-tailed spearman correlations were performed for the left and right hands. Statistical analysis was performed in SPSS (IBM, Armonk NY, USA) with a significance level of alpha < 0.05.

#### Motor cortex activation

A mixed effects model was performed comparing the VR BBT condition with 20 factors (all measurement channels) to the Physical BBT condition with 20 factors (all measurement channels) using beta values and fixing for participant specific error. A Benjamini–Hochberg statistical correction was applied to account for multiple comparisons. Statistical analyses were performed in MATLAB R2018a (MathWorks, Natick MA, USA) with statistical significance set to alpha < 0.05 and corrected to q < 0.05.

#### Neuromuscular activation

The Shapiro–Wilk test was used to determine the normality of neuromuscular activation variables. The following variables failed the test for normality, wrist extensors (p = 0.023) and bicep (p = 0.005) for the left hand manual condition, bicep (p = 0.005) for the right hand manual condition, wrist flexors (p = 0.039), wrist extensors (p = 0.043) and bicep (p = 0.001) for the left hand VR condition, wrist flexors (p = 0.002), bicep (p = 0.004), and triceps (p = 0.021) for the right hand VR condition. Two-tailed Wilcoxon signed rank tests were performed using EMG peaks for each muscle comparing the physical BBT condition to the VR BBT condition. Statistical analyses were performed in SPSS. With a significance level of alpha < 0.05.

## Data Availability

The data is available upon reasonable request from the corresponding author.
